# Surface-modified Ba(Zr_0.3_Ti_0.7_)O_3_ nanofibers by polyvinylpyrrolidone filler for poly(vinylidene fluoride) composites with enhanced dielectric constant and energy storage density

**DOI:** 10.1038/srep26198

**Published:** 2016-05-17

**Authors:** Shaohui Liu, Shuangxi Xue, Shaomei Xiu, Bo Shen, Jiwei Zhai

**Affiliations:** 1 Key Laboratory of Advanced Civil Engineering Materials of Ministry of Education, Functional Materials Research Laboratory, School of Materials Science & Engineering, Tongji University, 4800 Caoan Road, Shanghai 201804, China; 2 School of Science, Henan Institute of Engineering, Zhengzhou 451191, China

## Abstract

Ferroelectric-relaxor behavior of Ba(Zr_0.3_Ti_0.7_)O_3_ nanofibers (BZT NF) with a large aspect ratio were prepared via electrospinning and surface modified by PVP as dielectric fillers. The nanocomposite flexible films based on surface modified BZT NF and polyvinylidene fluoride (PVDF) were fabricated via a solution casting. The results show that the surface-modified BZT NF fillers are highly dispersed and well integrated in the PVDF nanocomposites. The nanocomposites exhibit enhanced dielectric constant and reduced loss tangents at a low volume fraction of surface-modified BZT NF. The polymer nanocomposites maintain a relatively high breakdown strength, which is favorable for enhancing energy storage density in the nanocomposites. The nanocomposite containing of 2.5 vol. % of PVP modified BZT NF exhibits energy density as high as 6.3 J/cm^3^ at 3800 kV/cm, which is more than doubled that of the pure PVDF of 2.8 J/cm^3^ at 4000 kV/cm. Such significant enhancement could be attributed to the combined effects of the surface modification and large aspect ratio of the BZT NF. This work may provide a route for using the surface modified ferroelectric-relaxor behavior of ceramic nanofibers to enhance the dielectric energy density in ceramic-polymer nanocomposites.

Compared with other energy storage devices (e.g., batteries, fuel cells), high energy density polymer capacitors possess the advantage of high power density due to the fast charge and discharge capability^1^,^2^,^3^,^4^,^5^,^6^,^7^,^8^. High energy density polymer capacitors have been attracting an increasingly fundamental and practical interest for their broad range of applications in electronic and electrical systems for energy pulse and power conditioning^9^,^10^,^11^,^12^,^13^. The energy density of dielectric materials is defined as 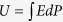 where *E* is electric field and *P* is polarization. Actually, the traditional dielectric materials, such as organic polymers and inorganic ceramics, fail to meet the rigorous requirements of advanced capacitors. Ferroelectric ceramics generally suffer from brittleness, low dielectric breakdown strength, and processing difficulty despite the high dielectric constant^13^,^14^,^15^. While, most polymers, including PVDF, BOPP, possess excellent electric breakdown properties, good flexibility and easy to process, but are limited by their low dielectric constants, which is usually less than 10. To overcome the disadvantages of either polymers or ceramics, there has been much effort to prepare polymer nanocomposites comprising organic polymers and inorganic ceramics^16^. The polymer nanocomposites combine both the advantages of polymers and ceramics (e.g., the high dielectric constant of the ceramic fillers and the high breakdown strength of polymers) and can exhibit high energy storage density. Ceramic-polymer nanocomposites usually exhibit good manufacturability, low cost and reduced weight, which is attractive for energy storage applications^17^,^18^,^19^.

By integrating the ferroelectric ceramics into the polymer matrix, novel nanocomposites have a high dielectric constant, energy storage density and good flexibility. Currently, BaTiO_3_, as a free lead material which is environmentally friendly, plays an important role in preparing the ceramic-polymer nanocomposite to improve the dielectric properties of polymer^20^,^21^. However, the application in certain areas of pure BaTiO_3_ ceramics is limited because of the narrow working temperature-stable range and high loss tangent. And being ferroelectric, they are defined by high remnant polarization, which leads to limited energy discharge. While the optimizing procedure leads to the selection of materials that have high maximum polarization (*P*_*max*_), low remnant polarization (*P*_*rem*_), high breakdown strength and Curie temperature well below the working temperature. Relaxor ferroelectrics have high *P*_*max*_, lower *P*_*rem*_ and slim hysteresis loop, which makes them promising candidate materials used for the energy storage ceramic capacitors. Ba(Zr_x_Ti_1−x_)O_3_ can be tuned to obtain a desired ferroelectric relaxor behavior by varying the stoichiometry^22^. After Zr^4+^ is introduced into BaTiO_3_, Zr^4+^ replaces Ti^4+^ in the crystal lattice. Thus, the dielectric constant maximum at the Curie point is broadened and shifted to around room temperature, resulting in a higher dielectric constant and much lower temperature coefficient of capacitance than pure BaTiO_3_. In the Ba(Zr_x_Ti_1−x_)O_3_ solid solution, transition occurs from the ferroelectric behavior to the ferroelectric relaxor behavior when the ratio of Zr/Ti is 0.27/0.73 and shows little hysteresis behavior at room temperature and above. The use of ferroelectric ceramics fillers such as Ba(Zr_0.3_Ti_0.7_)O_3_ (BZT) can provide a high dielectric constant while eliminating the remnant polarization and ultimately the efficiency of the capacitor.

The shape of the fillers and the interface between the fillers and the polymer are the important factors determining the energy storage density of the nanocomposites. A micromechanical model and experimental efforts by Andrews^23^ and Song^24^, have shown that nanocomposites filled with the higher aspect ratio exhibit greater dielectric constants than the composites with spherical at the same volume fraction. Moreover, phase-field modeling and experimental results all indicate that the orientation of large aspect ratios in ceramic fillers perpendicular to the direction of an external electric field is favorable for mitigating local field concentration in the nanocomposite and leads to higher breakdown strength^25^. The fillers with large aspect ratios is to achieve universally desirable dielectric properties (large dielectric constant and high breakdown strength) necessary for large energy storage density in dielectrics. At the same time, the interface between the fillers and the polymer matrix plays an important role in the energy storage nanocomposites^26^. The surface modification could facilitate the dispersion of the fillers in the polymer matrix and chain with the polymer matrix by chemical bonds in the interfacial layer, which can improve the energy storage density of the nanocomposites. Recently, many works about one dimensional perovskite fillers into the construction of nanocomposites have been reported. Such as Tang showed that significantly enhanced extractable energy storage densities are achieved in the PVDF matrix nanocomposites by filled with surface-modified Ba_0.2_Sr_0.8_TiO_3_ nanowires^27^. The surface-modified nanowires with coupling agent can improve the nanowires dispersion and enhance the interface adhesion. However, the price of coupling agent is high and the absorbed coupling agent on the filler surfaces may lead to high leakage current and high dielectric loss.

Up to now, there are few reports on the preparation of relaxor ferroelectrics BZT NF by employing surface modified by PVP as fillers in PVDF-based nanocomposites. In this study, we report the fabrication of BZT NF with a large aspect ratio via electrospinning and surface modified by PVP as dielectric fillers in PVDF-based nanocomposites. The effects of BZT NF on the microstructure and dielectric properties are discussed. The BZT NF with high dielectric constant and large aspect ratios give rise to the increased dielectric constant and breakdown strength of the nanocomposite at a small loading volume, which afford significantly enhanced energy storage density.

## Experimental process

The BZT NF prepared via electrospinning were employed as dielectric fillers in PVDF-based composites. Barium acetate (99.0%, Alfa Aesar) was dissolved in acetic acid and stirred for 1 h. Stoichiometric quantities of titanium (IV) n-butoxide (99.0%, Alfa Aesar) and zirconium isopropoxide was dissolved in 8 mL of acetylacetone and stirred for 1 h. Then, the dissolved solution of all compounds was mixed with a solution consisting of poly(vinyl pyrrolidone) (PVP, MW = 1 300 000) dissolved in ethanol (PVP: 2 g and ethanol: 3 ml). The mixture was stirred at room temperature for 1 h to achieve complete dissolution and mixing. Ultimately, a viscous sol solution was obtained to prepare for electrospinning. The precursor sol solution was loaded into a 10 ml plastic syringe with a syringe needle of which the internal diameter is 0.5 mm and electrospun with an applied electric field of 1.5 kV/cm, the as-electrospun fibers were then calcined at 900 °C for 3 h in air to get good crystallinity and remove PVP completely. PVP has recently been considered as a surface modification materials due to its strong interfacial adhesion strength to the surfaces including polymers and metal oxides. The BZT NF were dispersed into the ethanol solution of PVP and stirred for 10 h then separated by centrifugation, after dried at 80 °C for 12 h to obtain the PVP modified BZT NF.

**Figure 1:**
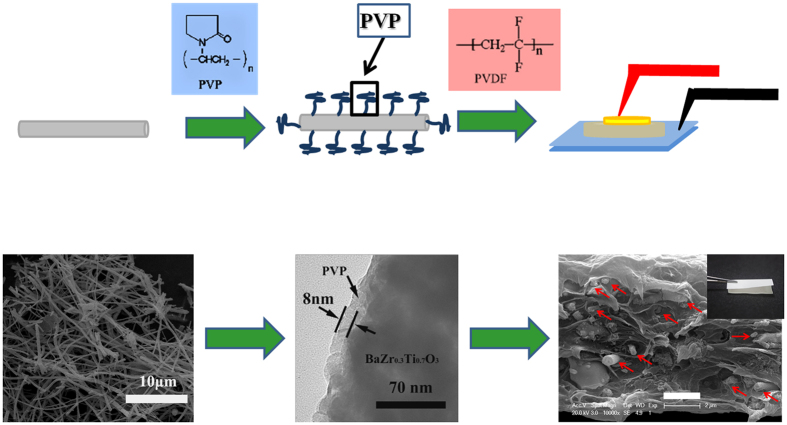
Schematic diagrams of the fabrication of surface-modified BZT NF/PVDF nanocomposites.

For the fabrication of the BZT NF/PVDF nanocomposites, the surface-modified BZT NF by PVP and PVDF powders (3F Co., China.) were proportionally dispersed in N, N-dimethylformamide (DMF) under vigorous stirring at 40 °C for 10 h to make it stable and homogenous. The suspension sol was cast onto an indium tin oxide (ITO) glass and dried under vacuum at 60 °C for 10 h for solvent volatilizing. The obtained films were heated at 200 °C for 10 min then immediately quenched in ice water. The final quenched films were dried at 40 °C for 24 h. The nanocomposite films were about 10 μm in thickness. Top Au electrodes were deposited onto the films using DC sputtering and a shadow mask for electrical measurements. Figure 1 shows the schematic diagram of the fabrication of surface-modified BZT NF/PVDF nanocomposites.

### Characterization

X-ray diffraction (XRD, Rigaku D-Max/2550VB+, Japan) was carried out to investigate the crystal structure of the samples with Cu-Kα radiation (λ = 1.5418 Å). Fourier-transform infrared spectroscopy (FT-IR) was performed with a Bruker Tensor 27 spectrometer over the range of 450–4000 cm^−1^ to determine the functionalization of the samples. X-ray photoelectron spectroscopy (XPS) was conducted using an ESCALAB 250Xi system X-ray photoelectron spectrometer equipment with Al Kα radiation (160 eV). High resolution transmission electron microscopy (HRTEM) images were obtained from a Philips CM200FEG instrument operated at an accelerating voltage at 200 kV. The samples for HRTEM characterization were prepared by dispersing a small amount of the powders in absolute ethanol and dropping the sample solutions onto carbon-coated copper grids and air-dried before measurement. Thermogravimetric analysis (TGA) was conducted by a NETZSCH STA449C instrument under air at a heating rate of 10 °C min^−1^. The morphology of the composites was performed by scanning electron microscopy (SEM, XL30-FEG, Philips, Netherlands). The dielectric properties of the composites were measured using an E4980A LCR meter (Agilent, Palo Alto, CA, USA) in a frequency range of 0.1 kHz to 2000 kHz at various temperatures. The displacement–electric field loops (D-E) and breakdown strength were obtained at 100 Hz in silicone oil by using a Premier II ferroelectric test system with high-voltage power supply (Precision Premier II; Radiant Technologies Inc, Albuquerque, New Mexico). The top gold electrodes with 2 mm in diameter and a thickness of 40 nm were sputtered on surfaces of the composites films using a shadow mask. The bottom electrode was the ITO glass.

## Results and Discussion

**Figure 2:**
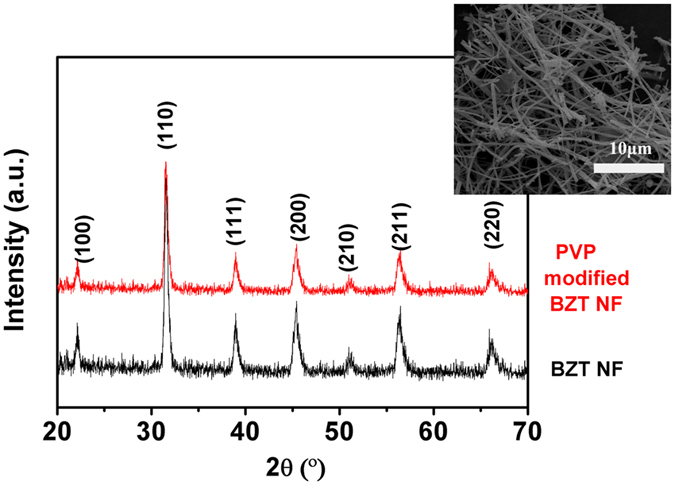
XRD patterns of BZT NF prepared via electrospinning and surface-modified BZT NF by PVP. SEM image is shown in the inset.

XRD patterns of BZT NF prepared via electrospinning and PVP modified BZT NF are given in Fig. 2. It is shown that the peaks at 2θ that correspond to 22° (100), 31° (110), 39° (111), 45° (200), and 56° (211) are assigned to BZT with a perovskite structure. No visible signal of the presence of secondary phases was observed. After modified by PVP, no changes in the crystal structure were found in the X-ray patterns of both PVP modified BZT NF and untreated BZT NF. SEM image reveals that the nanofibers have the large aspect ratios, i.e., diameters of 100–150 nm and lengths of tens of micrometers (inset of Fig. 2).

**Figure 3:**
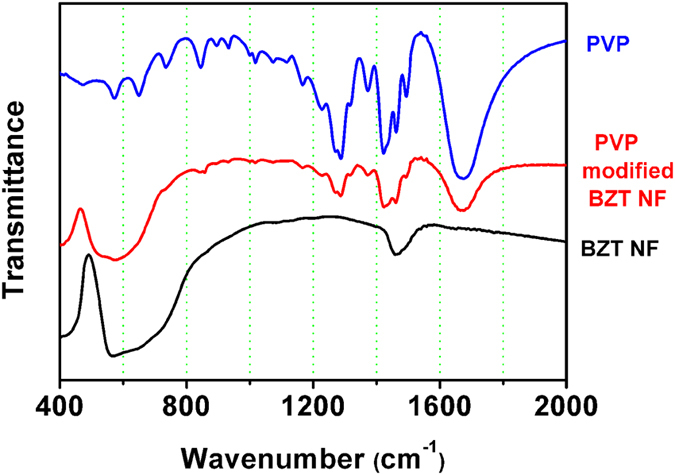
FTIR of untreated BZT NF, surface-modified BZT NF by PVP and PVP.

Figure 3 shows the FT-IR absorbance spectra of PVP modified BZT NF and untreated BZT NF. The band at 550 cm^−1^ corresponds to the Ti–O bond vibration in BZT NF. After the treatment of BZT NF with PVP, the absorption peak at 1663 cm^−1^ corresponds to the C-O stretching vibrations. In addition, the absorption peak located at 1435 cm^−1^ corresponds to aromatic amine C-N stretching vibrations. These peaks both are attributed to PVP^28^. The results of FTIR indicate that PVP has yielded a large coverage on the surface of BZT NF and formed robust bindings.

**Figure 4:**
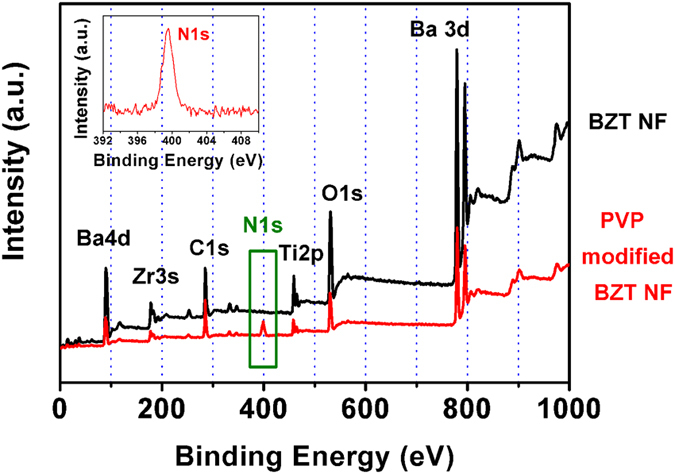
XPS spectra of BZT NF and surface-modified BZT NF. High-resolution XPS of N1s of surface-modified BZT NF is shown in the inset.

XPS was also used to confirm the PVP modified BZT NF. Figure 4 displays general XPS spectra of BZT NF and PVP modified BZT NF. The peak of N1s in the XPS spectra of BZT NF can hardly be found. After the treatment of BZT NF with PVP, the peak of N1s in the XPS spectra of PVP-modified BZT NF appears at 399.0 eV, corresponding to free -NH_2_. The high-resolution XPS spectrum of N1s of PVP-modified BZT NF is shown in the inset of Fig. 4. Strong signals of N1s elements appear in the general XPS spectrum of PVP-modified BZT NF, implying that the coating is derived from PVP. Due to the shielding by the PVP coatings on the surfaces of BZT NF, XPS signals of Ba3d, 4d electrons, Zr3s electrons, Ti 2p electrons from BZT were much reduced. In addition, the surface coating on the surface of the BZT NF was also confirmed by TGA data and TEM image.

**Figure 5:**
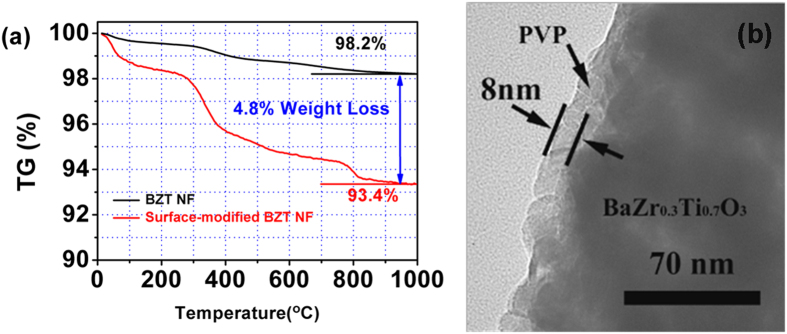
(**a**) TGA curve of modified and unmodified BZT NF and (**b**) PVP surface layers could be observed in the superimposed HRTEM image.

The differences in the TG data of BZT NF and surface-modified BZT NF present further evidence to prove the grafting of PVP on the surface of BZT NF (Fig. 5(a)). The weight loss below 100 °C is attributed to the deintercalation of water. The dramatic weight loss from 200 to 1000 °C results from the thermal decomposition of PVP. More weight loss of surface-modified BZT NF is observed at 1000 °C in comparison with the BZT NF. The differences in the TGA data of BZT NF and surface-modified BZT NF not only present further evidence to prove the grafting of PVP on the surface of BZT NF, but also give a PVP grafting fraction of about 4.8 wt% of the BZT NF. If we assume that the density of BZT NF is 6 g/cm^3^, the density of PVP layer is 1.144 g/cm^3^ and the average diameter of BZT NF is 120 nm and length is 10 μm, then we can estimate that the average PVP thickness on the surface of BZT NF is about 7 nm, estimated based on the combination of the weight loss during TG. This result is similar to that observed from TEM measurements. As shown in Fig. 5(b), PVP was uniformly coated on the surface of BZT NF with an average thickness of 8 nm revealing good coating effect.

**Figure 6:**
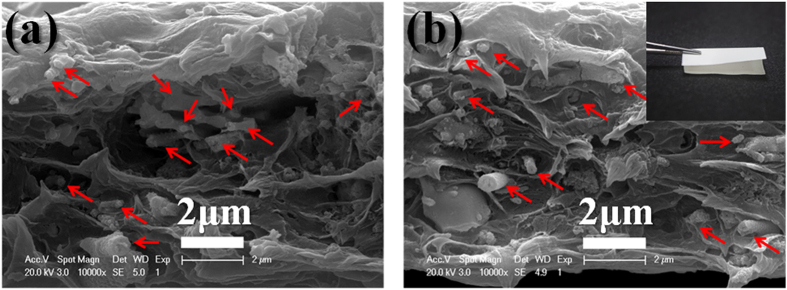
SEM images of the cross section of the PVDF nanocomposite films with the untreated BZT NF (**a**) and that with modified BZT NF (**b**) at a concentration of 5 vol%. The superior flexibility of the 5 vol% BZT NF is demonstrated by a macroscopic image shown in the inset of (**b**).

Figure 6 shows the cross-sectional SEM images of 5 vol.% BZT NF/PVDF and 5 vol.% PVP modified BZT NF/PVDF nanocomposites. The PVDF nanocomposites are mainly composed of PVDF and fiber with diameter about 200–300 nm, which is much bigger than the raw BZT NF. It is because the surface modified BZT NF have been successfully transferred to the PVDF and modified BZT NF and PVDF are tightly bound, which increase the diameter of the fiber fillers. The SEM images of the cross section of both nanocomposite suggest that the nanocomposite with the surface modified BZT NF shows less agglomeration than that with untreated BZT NF in Fig. 6. The surface modified BZT NF are well distributed in the PVDF polymer matrix and show little agglomeration in Fig. 6(b), meanwhile much more agglomeration is observed in the nanocomposite with untreated BZT NF in Fig. 6(a). There are much more small holes in nanocomposite films with the untreated BZT NF. The holes are due to great difference of intrinsic surface properties between the PVDF and untreated BZT NF. This result indicates there are much stronger interaction between modified BZT NF and PVDF polymer than that between untreated BZT NF and PVDF polymer. The nanofibers have been successfully transferred to the polymer matrix with minimum agglomeration and modified BZT NF tended to orient in the in-plane directions of the composite films. The superior flexibility of the nanocomposites with 5 vol% of modified BZT NF is demonstrated by a macroscopic picture shown in the inset of Fig. 6(b). It was found that the composite film still showed good flexibility at this loading level. This result could be explained by the good compatibility between functionalized nanofibers and the PVDF matrix resulted from the role performed by PVP.

**Figure 7:**
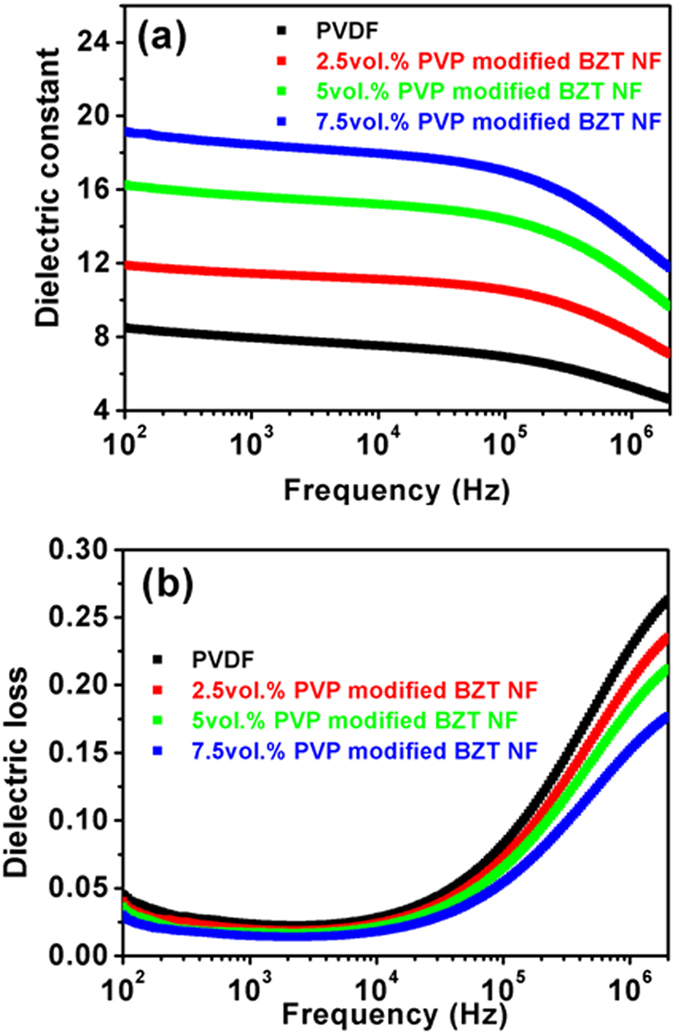
Frequency-dependence of (**a**) dielectric constant and (**b**) dielectric loss for composites with different volume fractions of PVP modified BZT NF.

The frequency dependence of dielectric constant and loss of pure PVDF and the nanocomposites are presented Fig. 7. It can be seen that the dielectric constant of the nanocomposites increases with increasing of PVP modified BZT NF content, and reaches up to 18.6 at small loading of 7.5 vol.% PVP modified BZT NF, which is mainly attributed to the large aspect ratio of BZT NF. Meanwhile, dielectric constant of pure PVDF and all composites decreases with frequency because the dipoles of filler and polymer cannot shift their orientation sufficiently fast when the frequency of the applied electric field exceeds the relaxation frequency^14^. In addition, the loss of the nanocomposites remains low with little variation at low frequency, followed by rapid increases at high frequency as a result of α_*a*_ relaxation which is associated with the glass transition of pure PVDF polymer^29^,^30^.

**Figure 8:**
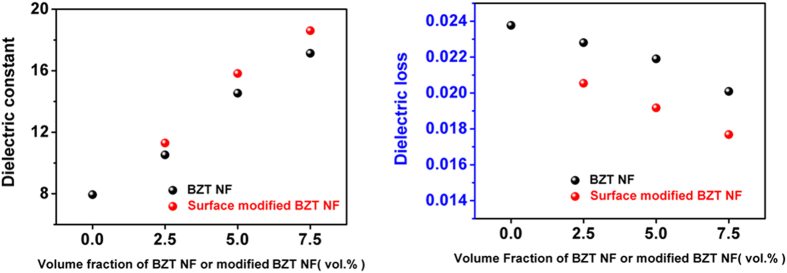
Dielectric constants (**a**) and dielectric loss (**b**) of BZT NF/PVDF and surface-modified BZT NF/PVDF nanocomposites loaded with various concentrations of fillers measured at 1 kHz.

The dielectric properties of the nanocomposite with the treated and untreated BZT NF have been investigated as a function of the BZT NF filler loading at a frequency of 1 kHz in Fig. 8. We can see that the dielectric constant of the nanocomposites increases with the BZT NF filler content. The maximum dielectric constant is 18.6 at 1 kHz at a small loading of 7.5 vol% PVP modified BZT NF, which is 2.35 times higher than that of the pure PVDF (7.9). This remarkable enhancement in the dielectric constant at such a small loading should be attributed to the higher dielectric constant of the BZT NF with the large aspect ratio^8^,^17^. In addition, the loss tangent of the nanocomposites remains quite low values for the nanocomposites with the treated BZT NF. Considering that the nanocomposites have a significantly increased dielectric constant and the lower dielectric loss than that of pure PVDF. Dielectric losses in a nanocomposite are related to the polarization mechanisms and the dipole relaxation associated with both materials, where the ceramic fillers normally display lower dissipation at a given frequency. The losses can also originate from the resonance of the polymer matrix at high frequency, ceramic fillers such as BaZr_0.3_Ti_0.7_O_3_ exhibit lower dielectric losses compared to the PVDF. So the reduction of the polymer content by increasing the ceramic filler loading reduces the loss tangent values. That may provide a promising method for using a small loading of surface-modified ceramic nanofibers for high dielectric constant polymer nanocomposites. As shown in Fig. 8, compared with nanocomposites with untreated BZT NF, the nanocomposite with the treated BZT NF at the same loading content not only shows a higher dielectric constant but also has a lower dielectric loss at a frequency of 1 kHz. Regarding the effect of composite microstructures on the dielectric property of nanocomposites, the good dispersion of fillers and the good interfacial adhesion between surfaces modified fillers and matrix are the important factors resulting in higher dielectric constant and lower dielectric loss. Previous TEM and XPS results indicate that PVP were introduced onto the surface of BZT NF. The surface modification benefits the homogenous distribution of BZT NF in the polymer matrix. In this case it will lead to higher dielectric constant and lower dielectric loss in the nanocomposites with the treated BZT NF.

**Figure 9:**
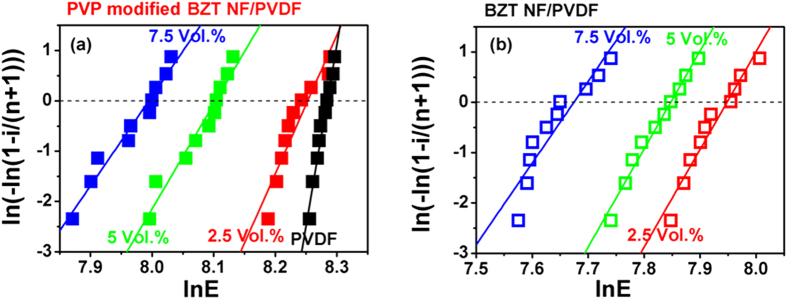
Weibull plots of breakdown strength of nanocomposites with different amount of untreated BZT NF and modified BZT NF.

The breakdown strength is the key parameter in determining the energy storage density of nanocomposites. The breakdown strength is analyzed using Weibull distribution, which are given by Equation (1) and Equation (2),


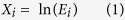



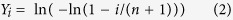


where *X*_*i*_ and *Y*_*i*_ are two parameters in Weibull distribution function, *E*_*i*_ is the specific breakdown voltage of each specimen in the experiments, *n* is the sum of specimens, and *i* is the serial number of specimens. The samples are arranged in ascending order of breakdown strength values so that *E*_1_ ≤ *E*_2_ ≤ ···*E*_*i*_··· ≤ *E*_*n*_. According to the Weibull distribution, *X*_*i*_ and *Y*_*i*_ have a linear relationship. The slope of the line is the shape parameter β, The mean breakdown strength could be extracted from points where the fitting lines intersect with the horizontal line through *Y*_*i*_ = 0. The Fig. 9a,b show the Weibull distribution of breakdown strength for the nanocomposite film loaded with various concentrations of the PVP modified BZT NF and untreated BZT NF. As seen from Fig. 9, all the plots show a relatively good linearity and the values of shape parameter β for the four samples all are higher than eight, thus a valid comparison of characteristic breakdown strength is possible.

**Figure 10:**
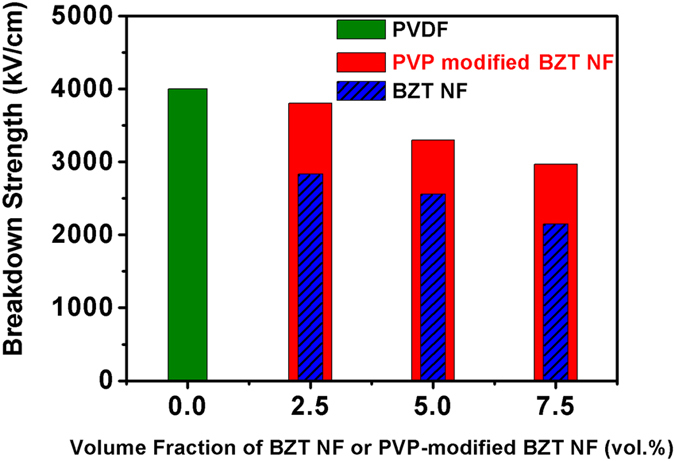
Breakdown strength of nanocomposites with different amount of untreated BZT NF and modified BZT NF.

Figure 10 shows the breakdown strength for nanocomposites with different amount of untreated BZT NF and modified BZT NF. The results illustrate that the nanocomposites with modified BZT NF maintain relatively high breakdown strength. That can be attributed to the surface modified BZT NF with large aspect ratio and tend to orient in the in-plane directions of the composite films during the solution cast process. When the electric field applied in the out-of-plane direction of the composite films, the susceptibility of the nanocomposites could be reduced by the modified BZT NF perpendicular to the external electric field, leading to a lower concentration of electric field in the polymer matrix^31^,^32^. The breakdown strength of both the nanocomposites with surface-modified BZT NF and those with untreated BZT NF decreases as the volume fraction of ceramic fillers gradually increases from 0 to 7.5 vol%. The breakdown strength shows much more sharp decrease at high filler concentration. The results can be explained by several reasons. First, when the dielectrics with high dielectric constant are added into the polymer, a highly inhomogeneous electric field is produced due to the large difference of dielectric constant between BZT NF fillers and PVDF polymer matrix, leading the electrical field in the polymer around the fillers much higher than the average electric field^33^. That leads to the reduction of the breakdown strength of the nanocomposites. Second, the agglomerations of BZT NF fillers and defects also have negative influences in breakdown strength of the nanocomposites. As the volume fraction of BZT NF fillers increases, much more agglomerations and defects such as voids are introduced into the nanocomposites. That also results in significant reduction of the breakdown strength of the nanocomposites, because electrical breakdown usually takes place in the weakest part of the materials. It should also be noted that greatly enhancement of electric breakdown strength has been attained on the nanocomposites with surface modified BZT NF comparing to those with untreated BZT NF. Especially, when the volume fraction of BZT NF is 7.5%, the electric breakdown strength of the nanocomposite with surface modified BZT NF increases to 2900 kV/cm, about 31% higher than that of the nanocomposite with untreated BZT NF. The enhancement of electric breakdown strength is directly attributed to the use of surface modified by PVP. The surface modification of the BZT NF by PVP contributes to the homogeneous distribution of the BZT NF in the polymer matrix, as shown in the inset SEM image of the cross-section of the nanocomposite film in Fig. 6.

**Figure 11:**
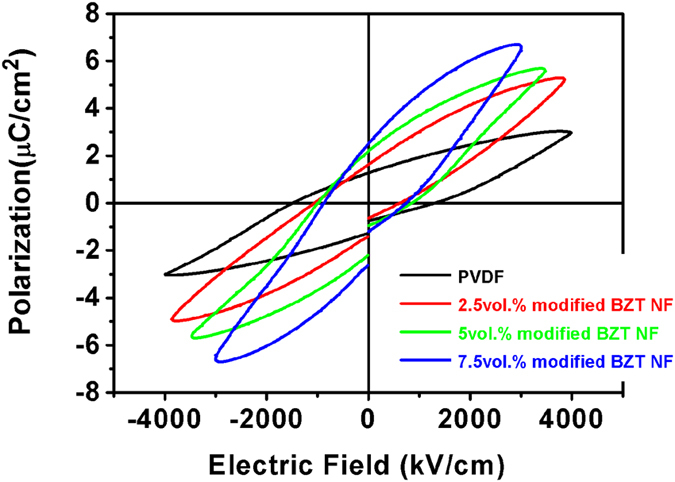
*P-E* loops for composites filled with different volume fraction of PVP modified BZT NF at 100 Hz and room temperature.

In order to calculate the energy density of the nanocomposites, *P-E* loops were measured from the PVDF-based nanocomposites with different amount of modified BZT NF at 100 Hz and room temperature as shown in Fig. 11. The incorporation of the surface-modified BZT NF into the PVDF polymer induces an improved *P*_*max*_, which was attributed to the high dielectric constant of BZT NF. While very minor increase in *P*_*rem*_ is observed. This result indicated that the use of relaxor ferroelectrics fillers such as Ba(Zr_0.3_Ti_0.7_)O_3_ provide a relatively high dielectric constant while eliminating the remnant polarization and ultimately the energy density of the nanocomposites. Furthermore, the *P-E* loops indicate that the electric displacement does not saturate at high electric field, which also benefits the energy density of the nanocomposites. The energy storage density of the nanocomposites can be calculated from the *P-E* loops based on the following integral 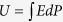, where *E* is the electric field and *P* is polarization. The energy storage density of nanocomposites loaded with various concentrations of modified BZT NF as function of electric field is presented in Fig. 11. The energy storage density of the composite with 2.5 vol.% reaches 6.3 J/cm^3^ at 3800 kV/cm, which is more than doubled as compared with the pure PVDF of 2.8 J/cm^3^ at 4000 kV/cm.

**Figure 12:**
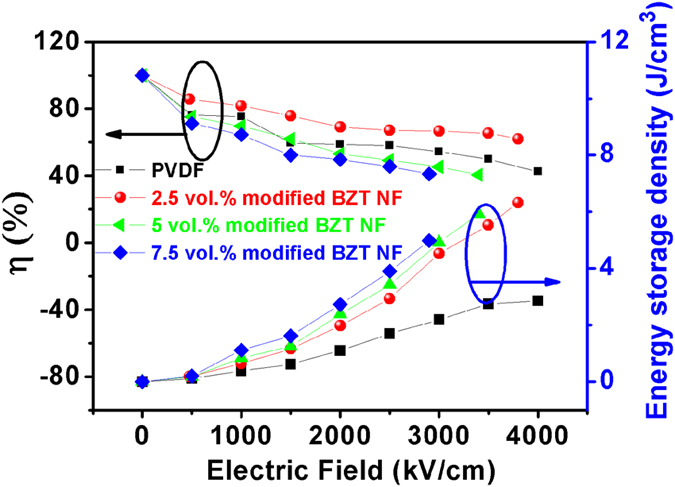
Energy storage density and discharge efficiency of nanocomposites with different volume fraction of PVP modified BZT NF.

For the application of energy storage capacitors in practice, both a high energy storage density and a high discharge efficiency (*η*) are desired. The discharge efficiency *η* could be calculated according to the formula: 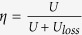, where *U*_*loss*_ is the energy loss density. The energy loss density was calculated by the numerical integration of closed area of the hysteresis loops. Figure 12 also gives the discharge efficiency of the nanocomposites with different PVP modified BZT NF concentrations as the function of the electric field. It is clearly shown that the discharge efficiency decreases with the applied electric filed, which is highly related to the leakage currents. As the concentration of the filler increases, the discharge efficiency of the nanocomposites decreases due to the larger hysteresis in the polarization. However, at electric fields below 1000 kV/cm, the discharge efficiency of the nanocomposites with 2.5 vol% PVP modified BZT NF is higher than 80% and still higher than 60% at an electric field of 3800 kV/cm, which is much higher than reported nanocomposites with BaTiO_3_ nanofibers. The reason of this is that the use of relaxor ferroelectrics such as Ba(Zr_0.3_Ti_0.7_)O_3_ have high *P*_*max*_, lower *P*_*rem*_ and slim hysteresis loop, which can provide a relatively high dielectric constant while eliminating the remnant polarization and ultimately the efficiency of the nanocomposites.

**Figure 13:**
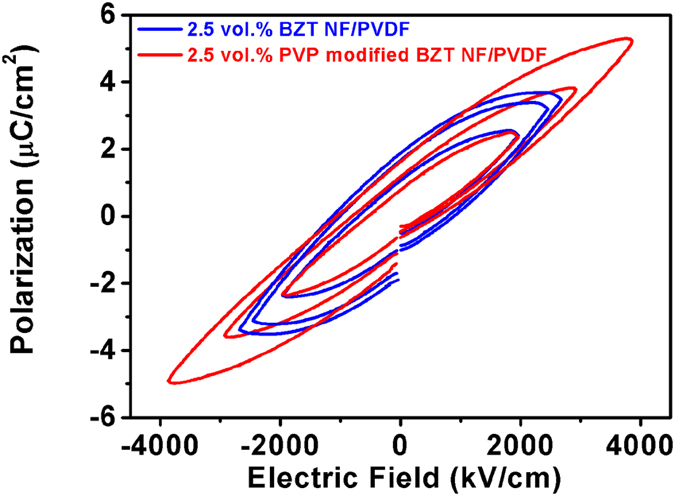
*P-E* loops under various applied electric field for 2.5 vol.% BZT NF/PVDF and 2.5 vol.% PVP modified BZT NF /PVDF nanocomposites.

**Figure 14:**
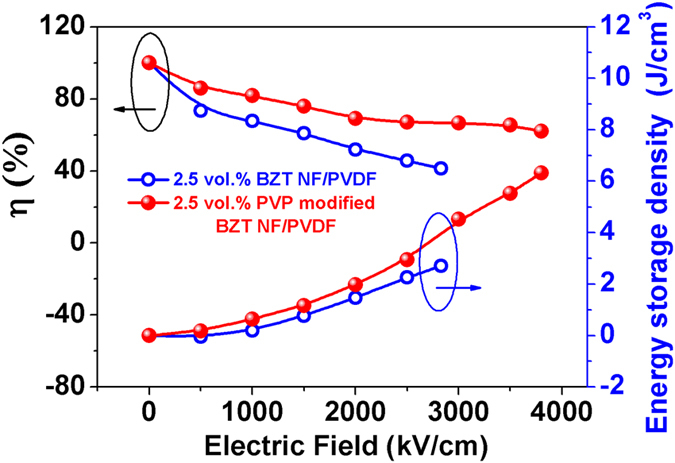
Energy efficiency and energy storage density under various applied electric field for 2.5 vol.% BZT NF/PVDF and 2.5 vol.% PVP modified BZT NF /PVDF nanocomposites.

Figure 13 gives the *P-E* loops of the nanocomposite with 2.5 vol.% BZT NF and that of 2.5 vol.% PVP modified BZT NF under various applied electric fields. The PVP modified BZT NF/PVDF nanocomposites exhibit narrow *P-E* loops, higher maximum polarization and much lower remnant polarization in comparison with the BZT NF/PVDF nanocomposites. Figure 14 gives the energy storage density and energy efficiency of the nanocomposite with the 2.5 vol.% surface modified BZT NF and untreated BZT NF under various applied electric fields. It can be seen that the energy storage density of the nanocomposite with the surface modified BZT NF is up to 6.3 J/cm^3^ at an electric field of 3800 kV/cm, about 133% higher than that with untreated BZT NF at an electric field of 2800 kV/cm. At an electric field of 2500 kV/cm the energy efficiency for the nanocomposites with 2.5 vol% surface modified BZT NF and BZT NF is 67.1% and 45.6% respectively. The former is 47.1% higher than the latter. These results clearly indicate the nanocomposite with the surface modified BZT NF exhibits much higher discharged energy-storage density and shows much higher energy efficiency in comparison with nanocomposite with the untreated BZT NF, which is due to the improvement of the homogeneity of the nanocomposite and the significant enhancement of the breakdown strength of the nanocomposites.

To elucidate the effect of the PVP shell layer thickness on the energy storage properties of 2.5 vol.% PVP modified BZT NF /PVDF nanocomposites, we compare the nanocomposites with different thickness of PVP on BZT NF. The results show that the energy storage properties of the nanocomposites with coating a 3 nm thick PVP BZT NF is 4.1 J/cm^3^ at an electric field of 3200 kV/cm. With a 8 nm thick PVP thickness coating on BZT NF, the energy storage density of the nanocomposites is up to 6.3 J/cm^3^ at an electric field of 3800 kV/cm, which is higher than that of the composite with coating a 3 nm thick PVP BZT NF. The energy storage density can be improved about 53.7% in comparison with the nanocomposite with coating a 3 nm thick PVP BZT NF. While, upon increasing the thickness of the PVP shells, With a 13 nm thick PVP thickness coating on BZT NF, the energy storage density of the nanocomposites is 4.9 J/cm^3^ at an electric field of 3400 kV/cm. The energy storage density of the composites is reduced. Perhaps two main factors are charge of the high energy storage properties in the composite with PVP modified. On the one hand, PVP acts as function groups which have established bindings with BZT NF and also have compatibility with PVDF polymer matrix, which contribute to the enhancement of the homogeneity and the energy storage properties of the nanocomposites, which afford significantly enhanced energy storage densities in the nanocomposites. On the other hand, a large amount of redundant residual PVP introduced into the nanocomposites results in the increase in the dielectric loss and dc leakage current density of the nanocomposites. That is unfavorable for improving the energy storage properties.

## Conclusions

BZT NF/PVDF composite flexible films with high dielectric constant and energy storage properties were fabricated through a solution casting method with surface modification of BZT NF by PVP. TEM results showed that PVP was uniformly coated on the surface of BZT NF with an average thickness of 8 nm. SEM results showed that the surface modification BZT NF were dispersed homogeneously in the polymer matrix. The enhanced of dielectric constant and reduced loss tangents has been achieved on the nanocomposites of low volume fractions of surface modified BZT NF. The energy storage density of 6.3 J/cm^3^ was obtained at 3800 kV/cm with 2.5 vol.% PVP modified BZT NF, which is 125% higher than that of the pure PVDF. Such significant enhancement is closely related to the combined effect of the large aspect ratio and the surface modification BZT NF. The results indicate a small loading of surface modification ceramics nanofiber can be used to enhance the energy storage density of nanocomposites, which may contribute to the development of ceramic-polymer nanocomposites for energy storage applications.

## Additional Information

**How to cite this article**: Liu, S. *et al*. Surface-modified Ba(Zr_0.3_Ti_0.7_)O_3_ nanofibers by polyvinylpyrrolidone filler for poly(vinylidene fluoride) composites with enhanced dielectric constant and energy storage density. *Sci. Rep.* **6**, 26198; doi: 10.1038/srep26198 (2016).
